# Supplementary material for: Estimating diagnostic uncertainty in artificial intelligence assisted pathology using conformal prediction
Source: Nat Commun. 2022 Dec 15;13:7761. doi: 10.1038/s41467-022-34945-8 (PMC9755280; doi:10.1038/s41467-022-34945-8)
Supplement: Supplementary file 1 — Supplementary Information [file 41467_2022_34945_MOESM1_ESM.pdf]

Supplementary appendix

Content

Content 1
Prostate pathology Imagebase reference panel 2
Supplementary figures 4
Supplementary tables 9

Prostate pathology Imagebase reference panel

Department of Oncology and Pathology, Karolinska Institutet, Stockholm, Sweden
Lars Egevad (panel leader and consortium representative)

Department of Pathology and Molecular Medicine, Wellington School of Medicine and Health Sciences, University of Otago, Wellington, New Zealand
Brett Delahunt

Aquesta Uropathology and University of Queensland, Brisbane, QLD, Australia
Hemamali Samaratunga

Department of Pathology and Molecular Medicine, Indiana University School of Medicine, Indianapolis, IN, USA
David J. Grignon

Laboratory Medicine Program, University Health Network, Toronto General Hospital, Toronto, ON, Canada
Andrew J. Evans & Theo van der Kwast

Barts Cancer Institute, Queen Mary University of London, London, UK
Daniel M. Berney

Department of Pathology, Taipei Veterans General Hospital, Taipei, Taiwan
Chin-Chen Pan

Institute of Pathology, University Hospital Bonn, Bonn, Germany
Glen Kristiansen

Department of Tissue Pathology and Diagnostic Oncology, Royal Prince Alfred Hospital and Central Clinical School, University of Sydney, Sydney, NSW, Australia
James G. Kench

Department of Cellular Pathology, Southmead Hospital, Bristol, UK
Jon Oxley

Department of Urology, Laboratory of Medical Research, University of São Paulo Medical School, São Paulo, Brazil
Katia R. M. Leite

Pathology and Laboratory Medicine Institute, Cleveland Clinic, Cleveland, OH, USA
Jesse K. McKenney

Department of Pathology, Yale University School of Medicine, New Haven, CT, USA
Peter A. Humphrey

Department of Pathology, Memorial Sloan-Kettering Cancer Center, New York, NY, USA
Samson W. Fine

Department of Surgical Pathology, School of Medicine, Aichi Medical University, Nagoya, Japan
Toyonori Tsuzuki

Department of Cellular Pathology, University Hospital of Wales, Cardiff, UK
Murali Varma

Department of Pathology and Laboratory Medicine, Tufts Medical Center, Boston, MA, USA
Ming Zhou

Hôpital Tenon, HUEP, AP-HP, UPMC Paris VI, Sorbonne Universities, Paris, France
Eva Comperat

Bostwick Laboratories, Orlando, FL, USA
David G. Bostwick

Department of Pathology, Medical College of Wisconsin, Milwaukee, WI, USA
Kenneth A. Iczkowski

Department of Anatomic Pathology, Cleveland Clinic Lerner College of Medicine, Cleveland Clinic, Cleveland, OH, USA
Cristina Magi-Galluzzi

Department of Laboratory Medicine and Pathobiology, University of Toronto, Toronto, ON, Canada
John R. Srigley

Department of Pathology, Jikei University School of Medicine, Tokyo, Japan
Hiroyuki Takahashi

Supplementary figures

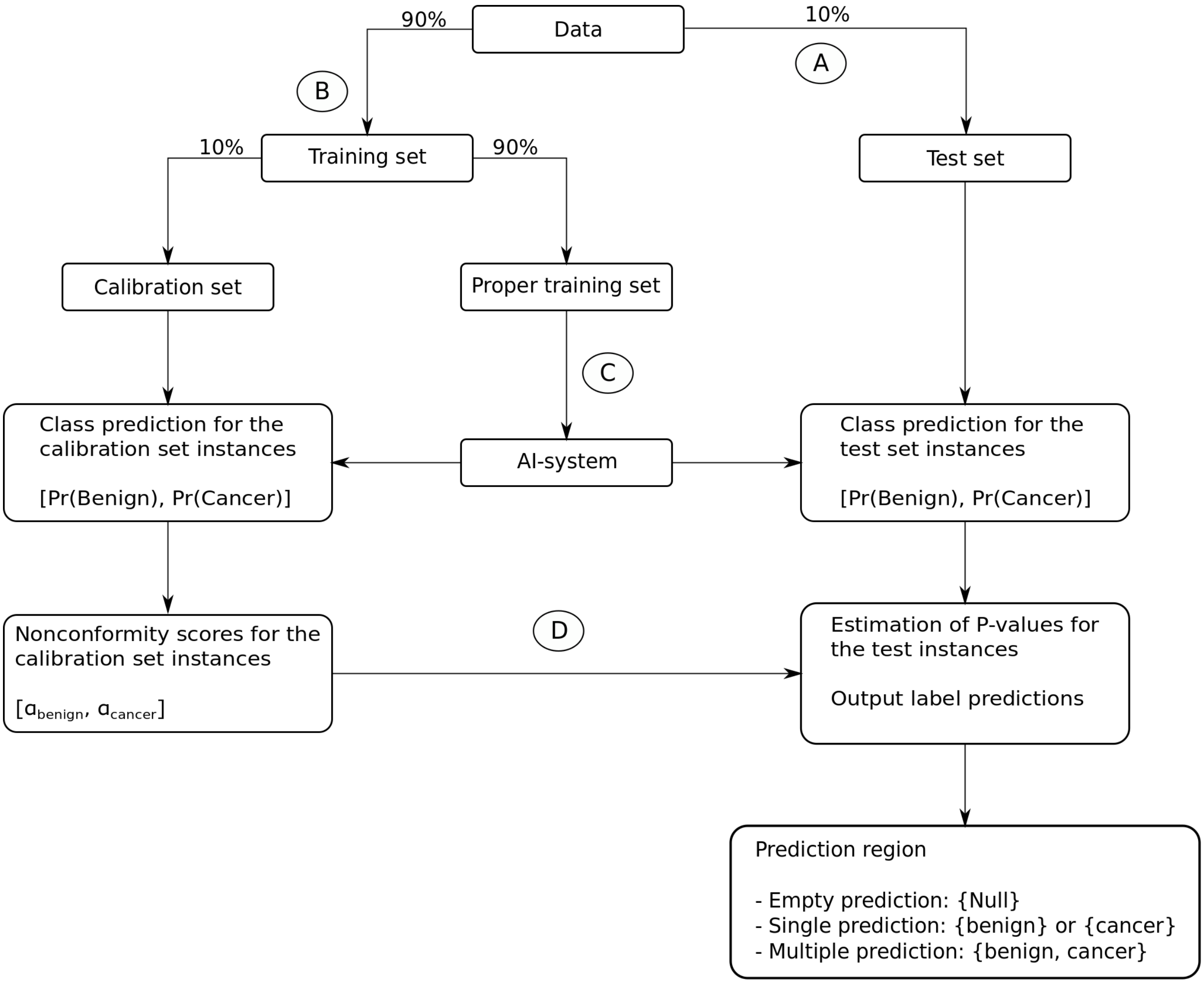

Supplementary Figure 1: Data utilization for training and evaluating the AI system and the conformal predictor. (A) 10% of the complete data was set aside as test data. (B) 90% of the training data was used to form the proper training set and 10% was used to form the calibration set. (C) The AI-system is trained on the proper training set and label predictions are made for each biopsy. (D) In the second step, the conformal predictor uses the calibration set to construct confidence regions for the newly generated predictions based on all the previously available examples. All data splits are made on a man level, to avoid biopsies from the same man to be present in both training and validation data.

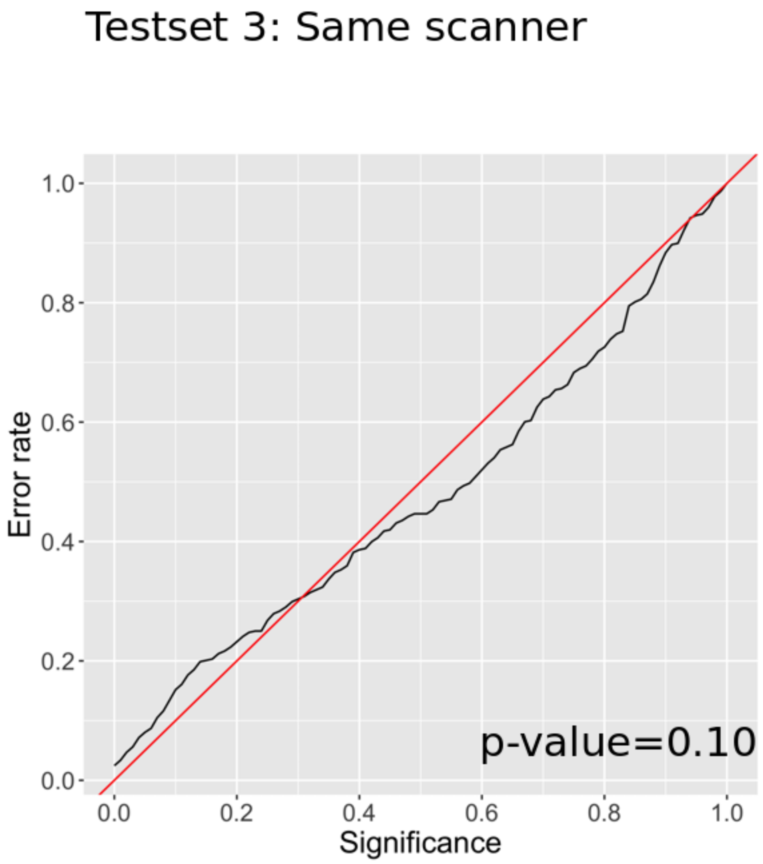

Supplementary Figure 2: Calibration plot of the observed prediction error (i.e. the fraction of true labels not included in the prediction region) on the y-axis and the prespecified significance level ε, i.e. the tolerated error rate. The conformal predictor is valid if the observed error rate does not exceed ε, i.e. the observed error rate should be close to the diagonal line, the tolerated error rate for all significance levels. The main advantage of conformal predictors is that they provide *valid* predictions when new examples are independent and identically distributed to the training examples. Biopsies from the STHLM3 study were digitized using two different scanners. We trained the AI system on only Aperio images and evaluated on a set of 449 biopsies that were scanned using both scanners. Thus, creating paired dataset comparison that singles out how model performance generalizes to a new scanner other than the one used to process the training data. The prediction regions were valid for the 449 biopsies when evaluated on same scanner used to process training data, as the prediction error is close to the tolerated error for all significance level (Kolmogorov-Smirnov $P > 0.05$).

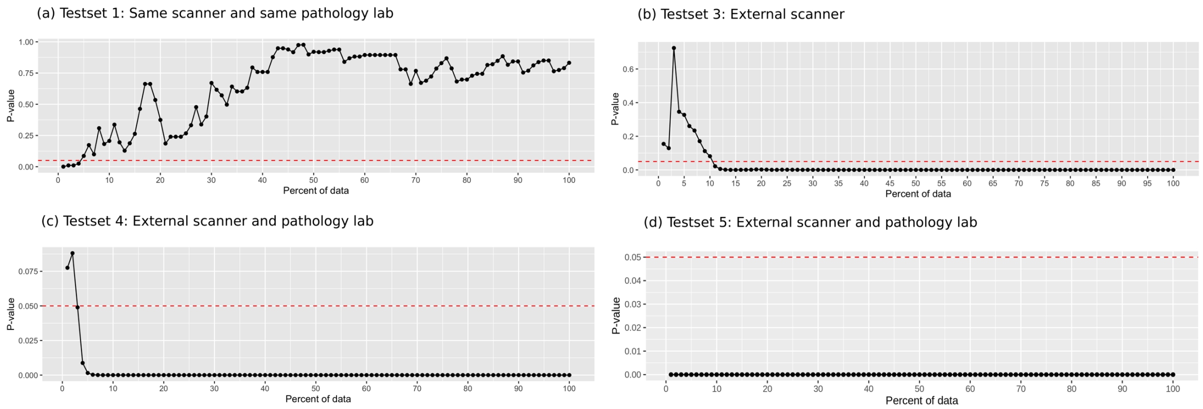

Supplementary Figure 3: Kolmogorov-Smirnov test of equality of the distribution of the nonconformity scores in the calibration set and the nonconformity scores was used to test the validity of the prediction regions for each test data. The figure illustrates how many observations that would be needed in order to detect systematic differences between training data and each external test data. This was done by estimating the power of the Kolmogorov-Smirnov test for Testsets 1, 3, 4 and 5 by repeated random sampling of sets of increasing size of conformity scores from the validation datasets. A p-value of less than 5% was considered statistically significant (two-sided). The red horizontal line refers to a 5% statistical significance level of the Kolmogorov-Smirnov test. Points below this reference line were considered as a statistically significant difference between training and test data. Less than 11% of the data from each Testset was needed in order to detect a decline in model performance on external data.

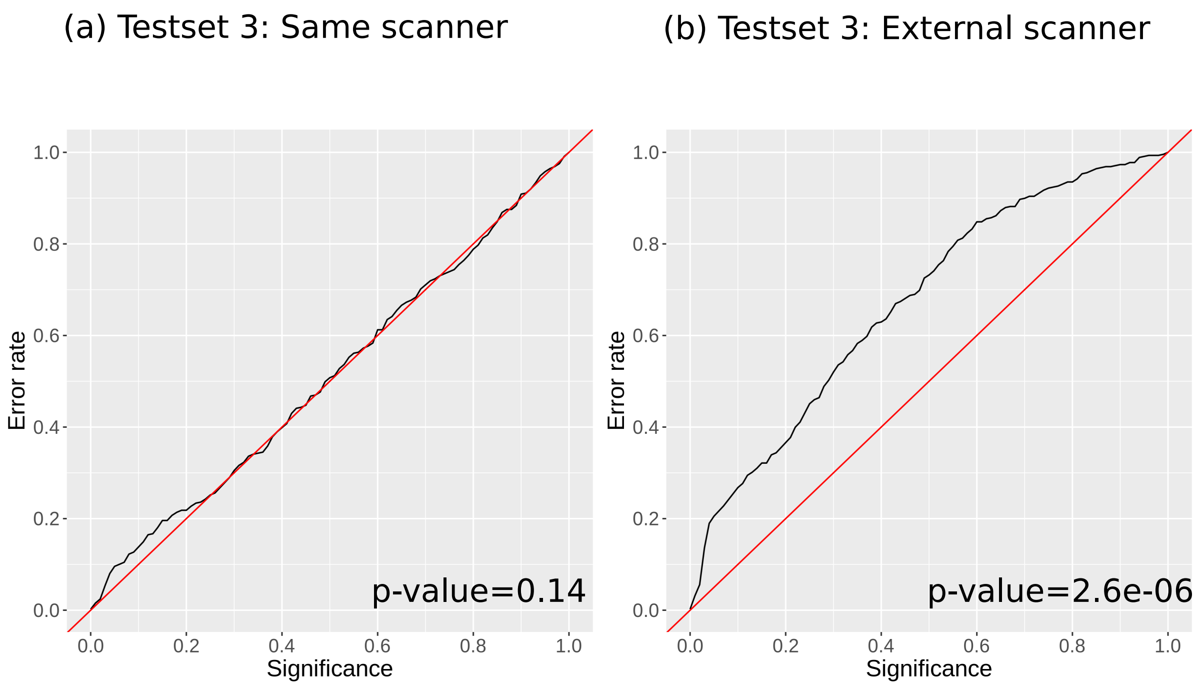

Supplementary Figure 4: Calibration plot of the prespecified significance level ε, i.e. the tolerated error rate (x-axis), plotted against the observed prediction error, i.e. the fraction of true labels not included in the prediction region (y-axis). The conformal predictor is valid if the observed error rate does not exceed ε, i.e. the observed error rate should be close to the diagonal line, the tolerated error rate for all significance levels. Biopsies from the STHLM3 study were digitized using two different scanners. Test set 3, a set of 449 slides, were scanned using both Hamamatsu and Aperio scanners to create a paired dataset, enabling direct comparison of how model performance generalizes to a new scanner other than the one used to process the training data. We trained the AI system on only Hamamatsu images and evaluated on the set of 449 biopsies scanned both on Hamamatsu and Aperio. We used the Kolmogorov-Smirnov test of equality of the distribution of the predictions in the calibration set and each test dataset to test the validity of the prediction regions. The null hypothesis was that the samples are drawn from the same distribution. A p-value of less than 5% was considered statistically significant (two-sided). Panel (a) shows that the prediction regions were valid when evaluated on the same scanner (Hamamatsu), as the prediction error is close to the tolerated error for all significance level (Kolmogorov-Smirnov $P > 0.05$). Panel (b): The prediction regions were non-valid when evaluating the same 449 slides on the Aperio scanner.

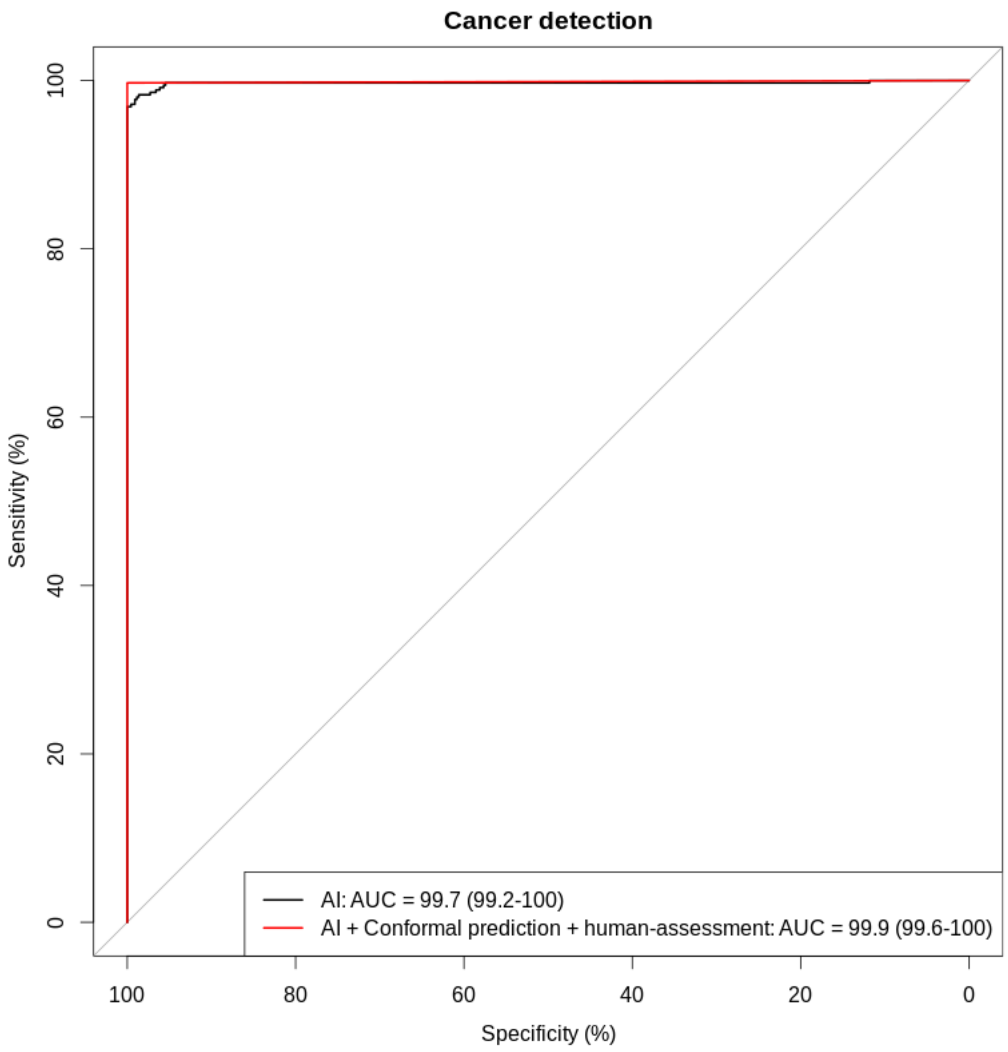

Supplementary Figure 5: Receiver operating characteristics curves and AUC for cancer detection on Test set 1 (n=794). AUC=area under the curve. The black line represents the ability of the AI system without conformal prediction to distinguish malignant from benign cores. The red line represents the following experimental approach: Firstly, point predictions are generated by the AI system. Secondly, the conformal predictor generates prediction intervals for the prediction at confidence level 99.9%, i.e. the true label of the case is guaranteed to be included in the prediction region with probability 99.9%. However, the predictions can be further divided into reliable single predictions and unreliable multi label predictions. Lastly, the unreliable predictions are assigned for human review, and the final grade is assigned by a pathologist. The standalone AI system achieved an AUC of 99.7% for cancer detection, while the experimental approach achieved an AUC of 99.9%. This shows how the error rate by AI models can be controlled via conformal prediction, and furthermore it illustrates the potential synergies of humans and machines working together to improve accuracy of prostate pathology.

Supplementary tables

Supplementary Table 1: Brief introduction to conformal prediction. Conformal prediction uses past experience to determine precise levels of confidence in new predictions. Given a user specified error probability ε, together with a prediction method (such as an AI system), it produces a set of labels that contains the true label with probability 1 - ε. Conformal prediction can be applied to any prediction algorithm. Constructing a conformal predictor involves the following steps: *Step 1: Select nonconformity measure***.** A nonconformity measure is a way of measuring how different a new example (i.e. an example for which we want to make predictions) is from previous examples. A simple nonconformity measure for classification problems is 1 minus the predicted probability of a class label. Given a nonconformity measure and a dataset, we can compute the nonconformity score α_i for each labeled example i=1,…,N. Each test example (with an unknown label) will then be assigned a potential label (e.g. {0}). For each possible potential label (e.g. {0,1} for binary classification), we calculate α (i.e. we test all potential labels for the example to find the one most conforming with the previous data). *Step 2: Compute p-values.* To measure how conforming a potential label for a test example N+1 is with previous data, we count how many α_i (i=1,…,N+1) that are equal to or larger than α_{N+1} of the test example, and divide by N+1. This ratio corresponds to the fraction of the training examples that are at least as conforming as the test example and is called the p-value. The larger the p-value, the more confident we are that the assigned label makes the example conform with previous data. *Step 3: Make predictions.* Given a user specified confidence level 1 - ε, the potential labels whose p-values are larger than ε will be accepted. The tables below schematically show predictions for 10 test examples at two different confidence levels (90% and 80%). With a higher confidence level, we get fewer error predictions (only test example 3 is incorrectly predicted), but more multiple predictions. At a lower confidence level, we get an additional error (test example 8), but fewer multiple predictions. We also get an empty prediction (test example 4), i.e. an example for which no p-value was larger than ε and a prediction could thus not be made. Conformal prediction thus enables us to only accept predictions with high confidence, such that the error rate can be kept low. The tradeoff is that the conformal predictor can output empty or multiple predictions, which identifies cases where the conformal predictor cannot assign reliable single predictions.

Test example	Predicted labels	True label	Prediction type	Test example	Predicted labels	True label	Prediction type
1	{0, 1}	0	Multiple	1	{0}	0	Single
2	{0}	0	Single	2	{0}	0	Single
3	{0}	1	Error	3	{0}	1	Error
4	{0}	0	Single	4	{}	0	Empty
5	{1}	1	Single	5	{1}	1	Single
6	{0, 1}	1	Multiple	6	{0, 1}	1	Multiple
7	{1}	1	Single	7	{1}	1	Single
8	{0, 1}	1	Multiple	8	{0}	1	Error
9	{0, 1}	0	Multiple	9	{0, 1}	0	Multiple
10	{0, 1}	1	Multiple	10	{1}	1	Single

Supplementary Table 2: Prediction regions on the ISUP Pathology Imagebase dataset (Test set 2) by ISUP score, n (%). The Imagebase dataset was independently graded by 23 uropathologists. The performance in ISUP grading was evaluated using the mode of the ISUP grades assigned by the 23 Imagebase uropathologists as ground truth.

**ISUP grade**							
Confidence		ISUP 1 (n=21)	ISUP 2 (n=32)	ISUP 3 (n=15)	ISUP 4 (n=8)	ISUP 5 (n=11)	All grades (n=87)
67%	Error, n (%)	10 (48%)	6 (19%)	4 (27%)	2 (25%)	5 (45%)	27 (31%)
	Empty, n (%)	0 (0)	0 (0)	0 (0)	0 (0)	0 (0)	0 (0)
	Single predictions, n (%)	11 (52%)	19 (59%)	7 (47%)	4 (50%)	2 (18%)	43 (49%)
	Multiple predictions, n (%)	0 (0)	7 (22%)	4 (27%)	2 (25%)	4 (36%)	17 (20%)
80%	Error, n (%)	6 (29%)	4 (12%)	4 (27%)	0 (0)	2 (18%)	16 (18%)
	Empty, n (%)	0 (0)	0 (0)	0 (0)	0 (0)	0 (0)	0 (0)
	Single predictions, n (%)	9 (43%)	10 (31%)	4 (27%)	4 (50%)	2 (18%)	29 (33%)
	Multiple predictions, n (%)	6 (29%)	18 (56%)	7 (47%)	4 (50%)	7 (64%)	42 (48%)

Supplementary Table 3: Prediction regions on the baseline test set (Testset 1). The predictions regions are evaluated against the grade assigned by an experienced uro-pathologist (L.E.) as ground truth. The rows show prediction regions assigned by the conformal predictor at confidence levels 80% and 67%, respectively. Percentages represent column percentages.

**Confidence: 80%**						
**Prediction regions, n (%)**	ISUP 1 (n=172)	ISUP 2 (n=62)	ISUP 3 (n=31)	ISUP 4 (n=41)	ISUP 5 (n=48)	All ISUP grades (n=354)
Empty	3 (2%)	2 (3%)	0 (0%)	1 (2%)	1 (2%)	7 (2%)
ISUP1	97 (56%)	8 (13%)	0 (0%)	0 (0%)	0 (0%)	105 (30%)
ISUP1 ISUP2	41 (24%)	14 (23%)	0 (0%)	0 (0%)	0 (0%)	55 (16%)
ISUP1 ISUP2 ISUP3	3 (2%)	1 (2%)	0 (0%)	0 (0%)	0 (0%)	4 (1%)
ISUP2	7 (4%)	8 (13%)	3 (10%)	1 (2%)	0 (0%)	19 (5%)
ISUP2 ISUP3	18 (10%)	16 (26%)	12 (39%)	2 (5%)	1 (2%)	49 (14%)
ISUP2 ISUP3 ISUP4	1 (1%)	1 (2%)	4 (13%)	1 (2%)	0 (0%)	7 (2%)
ISUP2 ISUP3 ISUP4 ISUP5	1 (1%)	4 (6%)	2 (6%)	1 (2%)	1 (2%)	9 (3%)
ISUP3	0 (0%)	3 (5%)	3 (10%)	3 (7%)	1 (2%)	10 (3%)
ISUP3 ISUP4	0 (0%)	1 (2%)	3 (10%)	1 (2%)	2 (4%)	7 (2%)
ISUP3 ISUP4 ISUP5	1 (1%)	4 (6%)	1 (3%)	10 (24%)	12 (25%)	28 (8%)
ISUP4	0 (0%)	0 (0%)	2 (6%)	6 (15%)	1 (2%)	9 (3%)
ISUP4 ISUP5	0 (0%)	0 (0%)	1 (3%)	14 (34%)	11 (23%)	26 (7%)
ISUP5	0 (0%)	0 (0%)	0 (0%)	1 (2%)	18 (38%)	19 (5%)
**Confidence: 67%**						
**Prediction regions, n (%)**	ISUP 1 (n=172)	ISUP 2 (n=62)	ISUP 3 (n=31)	ISUP 4 (n=41)	ISUP 5 (n=48)	All ISUP grades (n=354)
Empty	5 (3%)	2 (3%)	0 (0%)	2 (5%)	1 (2%)	10 (3%)
ISUP1	114 (66%)	11 (18%)	0 (0%)	0 (0%)	0 (0%)	125 (35%)
ISUP1 ISUP2	4 (2%)	3 (5%)	0 (0%)	0 (0%)	0 (0%)	7 (2%)
ISUP2	36 (21%)	20 (32%)	4 (13%)	1 (2%)	0 (0%)	61 (17%)
ISUP2 ISUP3	12 (7%)	17 (27%)	15 (48%)	1 (2%)	2 (4%)	47 (13%)
ISUP2 ISUP3 ISUP4	0 (0%)	0 (0%)	0 (0%)	1 (2%)	0 (0%)	1 (0%)
ISUP3	0 (0%)	5 (8%)	7 (23%)	4 (10%)	3 (6%)	19 (5%)
ISUP3 ISUP4	0 (0%)	2 (3%)	1 (3%)	7 (17%)	4 (8%)	14 (4%)
ISUP3 ISUP4 ISUP5	0 (0%)	2 (3%)	1 (3%)	4 (10%)	8 (17%)	15 (4%)
ISUP3 ISUP5	1 (1%)	0 (0%)	0 (0%)	0 (0%)	0 (0%)	1 (0%)
ISUP4	0 (0%)	0 (0%)	2 (6%)	12 (29%)	2 (4%)	16 (5%)
ISUP4 ISUP5	0 (0%)	0 (0%)	1 (3%)	4 (10%)	7 (15%)	12 (3%)

ISUP5	0 (0%)	0 (0%)	0 (0%)	5 (12%)	21 (44%)	26 (7%)
