# Supplementary material for: Estimating diagnostic uncertainty in artificial intelligence assisted pathology using conformal prediction
Source: Nat Commun. 2022 Dec 15;13:7761. doi: 10.1038/s41467-022-34945-8 (PMC9755280; doi:10.1038/s41467-022-34945-8)
Supplement: Supplementary file 3 — Reporting Summary [file 41467_2022_34945_MOESM3_ESM.pdf]

nature portfolio

Corresponding author(s): Henrik Olsson

Last updated by author(s): Oct 5, 2022

Reporting Summary

Nature Portfolio wishes to improve the reproducibility of the work that we publish. This form provides structure for consistency and transparency in reporting. For further information on Nature Portfolio policies, see our Editorial Policies and the Editorial Policy Checklist.

Statistics

For all statistical analyses, confirm that the following items are present in the figure legend, table legend, main text, or Methods section.

n/a	Confirmed	
☐	☒	The exact sample size (n) for each experimental group/condition, given as a discrete number and unit of measurement
☐	☒	A statement on whether measurements were taken from distinct samples or whether the same sample was measured repeatedly
☐	☒	The statistical test(s) used AND whether they are one- or two-sided<br>*Only common tests should be described solely by name; describe more complex techniques in the Methods section.*
☐	☒	A description of all covariates tested
☐	☒	A description of any assumptions or corrections, such as tests of normality and adjustment for multiple comparisons
☐	☒	A full description of the statistical parameters including central tendency (e.g. means) or other basic estimates (e.g. regression coefficient) AND variation (e.g. standard deviation) or associated estimates of uncertainty (e.g. confidence intervals)
☐	☒	For null hypothesis testing, the test statistic (e.g. F, t, r) with confidence intervals, effect sizes, degrees of freedom and P value noted<br>*Give P values as exact values whenever suitable.*
☒	☐	For Bayesian analysis, information on the choice of priors and Markov chain Monte Carlo settings
☐	☒	For hierarchical and complex designs, identification of the appropriate level for tests and full reporting of outcomes
☒	☐	Estimates of effect sizes (e.g. Cohen's d, Pearson's r), indicating how they were calculated

Our web collection on statistics for biologists contains articles on many of the points above.

Software and code

Policy information about availability of computer code

Data collection	The training and testing slides were digitized using either a Hamamatsu C9600-12 scanner and NDP.scan v. 2.5.86 software (Hamamatsu Photonics, Hamamatsu, Japan), or Aperio ScanScope AT2 scanner and Aperio Image Library v. 12.0.15 software (Leica Biosystems, Wetzlar, Germany).
Data analysis	Deep learning models were implemented in Python (version 3.6.9) using TensorFlow (version 2.6.2). Conformal prediction was implemented in R (version 4.0.0). The code for the analysis is available at https://github.com/heolss/Conformal_analyses, and in the Zenodo database at https://doi.org/10.5281/zenodo.7147740.

For manuscripts utilizing custom algorithms or software that are central to the research but not yet described in published literature, software must be made available to editors and reviewers. We strongly encourage code deposition in a community repository (e.g. GitHub). See the Nature Portfolio guidelines for submitting code & software for further information.

Data

Policy information about availability of data

All manuscripts must include a data availability statement. This statement should provide the following information, where applicable:

- Accession codes, unique identifiers, or web links for publicly available datasets
- A description of any restrictions on data availability
- For clinical datasets or third party data, please ensure that the statement adheres to our policy

All data underlying this article cannot be shared publicly for the privacy of individuals that participated in the STHLM3 diagnostic study. They can be made available through contact with ME under research collaboration and data sharing agreements. Source data are provided with this paper. Anonymized demo versions of the datasets that are used for the main analyses in the manuscript are available at https://github.com/heolss/Conformal_analyses, and in the Zenodo database, at

https://doi.org/10.5281/zenodo.7147740. Anonymized training data are available as part of the PANDA challenge (https://www.kaggle.com/c/prostate-cancer-grade-assessment and Bulten et al. Nature Medicine, 2022).

Field-specific reporting

Please select the one below that is the best fit for your research. If you are not sure, read the appropriate sections before making your selection.

- [x] Life sciences
- [ ] Behavioural & social sciences
- [ ] Ecological, evolutionary & environmental sciences

For a reference copy of the document with all sections, see nature.com/documents/nr-reporting-summary-flat.pdf

Life sciences study design

All studies must disclose on these points even when the disclosure is negative.

Sample size	No formal sample size calculation was performed. This was motivated by the fact that we do not have a specific null hypothesis to test. We digitized all cases enrolled in the STHLM3 study with GS 5+5 and 4+4 plus a stratified random selection of the other GS including benign subjects. The size of the test set was chosen as a tradeoff between leaving enough data for efficient training and for achieving high enough precision in the evaluation.
Data exclusions	No data were excluded from the analyses.
Replication	All attempts at replication of the experimental findings were successful. Accuracy in cancer detection and ISUP grading was evaluated in the internal test dataset, and grading performance was also evaluated on 87 biopsies digitized biopsies from the Imagebase database. The accuracy was similar in both test datasets. Furthermore, results on the Imagebase database showed that the uncertainty in the grading by the AI system closely approximated the uncertainty associated with the grades by the pathologist panel. The ability of the conformal predictor to detect unreliable predictions caused by differences between training and test data was evaluated and confirmed in total in four test datasets. The conformal predictor was able to detect unreliable predictions on these datasets, due to changes in tissue preparation techniques in different laboratories, digitization utilizing different digital pathology scanners, and the presence of atypical prostatic tissue, such as variants of prostatic adenocarcinoma and benign mimics of cancer.
Randomization	The study data were randomly split into a proper training set of 6951 biopsies from 1069 men, the calibration set consisting of 837 biopsies from 123 men, and a test set of 794 biopsies from 123 men. The training set was used to train the deep learning models and the calibration set was used for construction of the conformal p-values. We employed a collection of six different datasets (numbered 1-6 in the manuscript) comprising, in total, 3059 digitized biopsies for the evaluation of the AI system and conformal predictor.
Blinding	The group allocation of the main training and test split was based on random sampling, stratified on a man level to keep data independent in training and test data. In addition, external validation sets were used. The biopsies in the six test datasets were blinded to the investigators during model development and were excluded from any analysis until the final evaluation.

Reporting for specific materials, systems and methods

We require information from authors about some types of materials, experimental systems and methods used in many studies. Here, indicate whether each material, system or method listed is relevant to your study. If you are not sure if a list item applies to your research, read the appropriate section before selecting a response.

Materials & experimental systems

n/a	Involved in the study	
x		Antibodies
x		Eukaryotic cell lines
x		Palaeontology and archaeology
x		Animals and other organisms
	x	Human research participants
x		Clinical data
x		Dual use research of concern

Methods

n/a	Involved in the study	
x		ChIP-seq
x		Flow cytometry
x		MRI-based neuroimaging

Human research participants

Policy information about studies involving human research participants

Population characteristics	Between May 28, 2012, and Dec 30, 2014, the prospective, population-based, screening-by-invitation STHLM3 study (ISRCTN84445406) evaluated a diagnostic model for prostate cancer in men aged 50–69 years residing in Stockholm, Sweden. STHLM3 participants had 10–12-core transrectal ultrasound-guided systematic biopsies if they had prostate-specific antigen (PSA) concentration of 3 ng/mL or more or a Stockholm3 test score of 10% or more.
Recruitment	In total a random selection of 145,905 men were invited to the study, 59,149 men were included to the study and 7,417 men were biopsied. The STHLM3 study was performed in Sweden, and most participants were of northern European descent. The use of the AI system and conformal predictor have not been validated in other ethnic groups.
Ethics oversight	The study protocol was approved by the Stockholm regional ethics committee (permits 2012/572-31/1, 2012/438-31/3, and 2018/845-32), the Regional Committee for Medical and Health Research Ethics (REC) in Western Norway (permits REC/Vest 80924, REK 2017/71). Informed consent was provided by the participants in the Swedish dataset. For the other datasets, informed consent was waived due to the usage of de-identified prostate specimens in a retrospective setting.

Note that full information on the approval of the study protocol must also be provided in the manuscript.
