# Supplementary material for: Estimating diagnostic uncertainty in artificial intelligence assisted pathology using conformal prediction
Source: Nat Commun. 2022 Dec 15;13:7761. doi: 10.1038/s41467-022-34945-8 (PMC9755280; doi:10.1038/s41467-022-34945-8)
Supplement: Supplementary file 4 — Description of Additional Supplementary Files [file 41467_2022_34945_MOESM4_ESM.docx]

**Description of Additional Supplementary Files**

File Name: Supplementary Data 1

Description: Prediction regions on the ISUP Pathology Imagebase dataset (Test set 2). The cases in the database have been reviewed by the ISUP Imagebase panel consisting of 23 experienced urological pathologists. The rows show prediction regions assigned by the conformal predictor at confidence levels 80% and 67%, respectively. The predictions regions are evaluated against the mode assessment of ISUP grading by the Imagebase-panel (shown in the columns named “ISUP 1” to “ISUP 5”). We also compared the prediction regions by the conformal predictor against each individual panel member’s assigned ISUP grade. The prediction regions covered a median 65% of the individual votes by the 23 pathologists’ at confidence level 67% and a median 83% of panel votes at confidence level 80%. Percent of pathologists’ votes = percent of pathologist votes that are covered by the prediction regions. Percentages in other cells represent column percentages.
